# Host Subtraction, Filtering and Assembly Validations for Novel Viral Discovery Using Next Generation Sequencing Data

**DOI:** 10.1371/journal.pone.0129059

**Published:** 2015-06-22

**Authors:** Gordon M. Daly, Richard M. Leggett, William Rowe, Samuel Stubbs, Maxim Wilkinson, Ricardo H. Ramirez-Gonzalez, Mario Caccamo, William Bernal, Jonathan L. Heeney

**Affiliations:** 1 Lab of Viral Zoonotics, Department of Veterinary Medicine, University of Cambridge, Madingley Road, Cambridge, CB30ES, United Kingdom; 2 The Genome Analysis Centre (TGAC), Norwich Research Park, Norwich, NR47UH, United Kingdom; 3 Institute of Liver Studies, King's College Hospital, Denmark Hill, London, SE59RS, United Kingdom; Georgia Institute of Technology, UNITED STATES

## Abstract

The use of next generation sequencing (NGS) to identify novel viral sequences from eukaryotic tissue samples is challenging. Issues can include the low proportion and copy number of viral reads and the high number of contigs (post-assembly), making subsequent viral analysis difficult. Comparison of assembly algorithms with pre-assembly host-mapping subtraction using a short-read mapping tool, a k-mer frequency based filter and a low complexity filter, has been validated for viral discovery with Illumina data derived from naturally infected liver tissue and simulated data. Assembled contig numbers were significantly reduced (up to 99.97%) by the application of these pre-assembly filtering methods. This approach provides a validated method for maximizing viral contig size as well as reducing the total number of assembled contigs that require down-stream analysis as putative viral nucleic acids.

## Introduction

**Fig 1:**
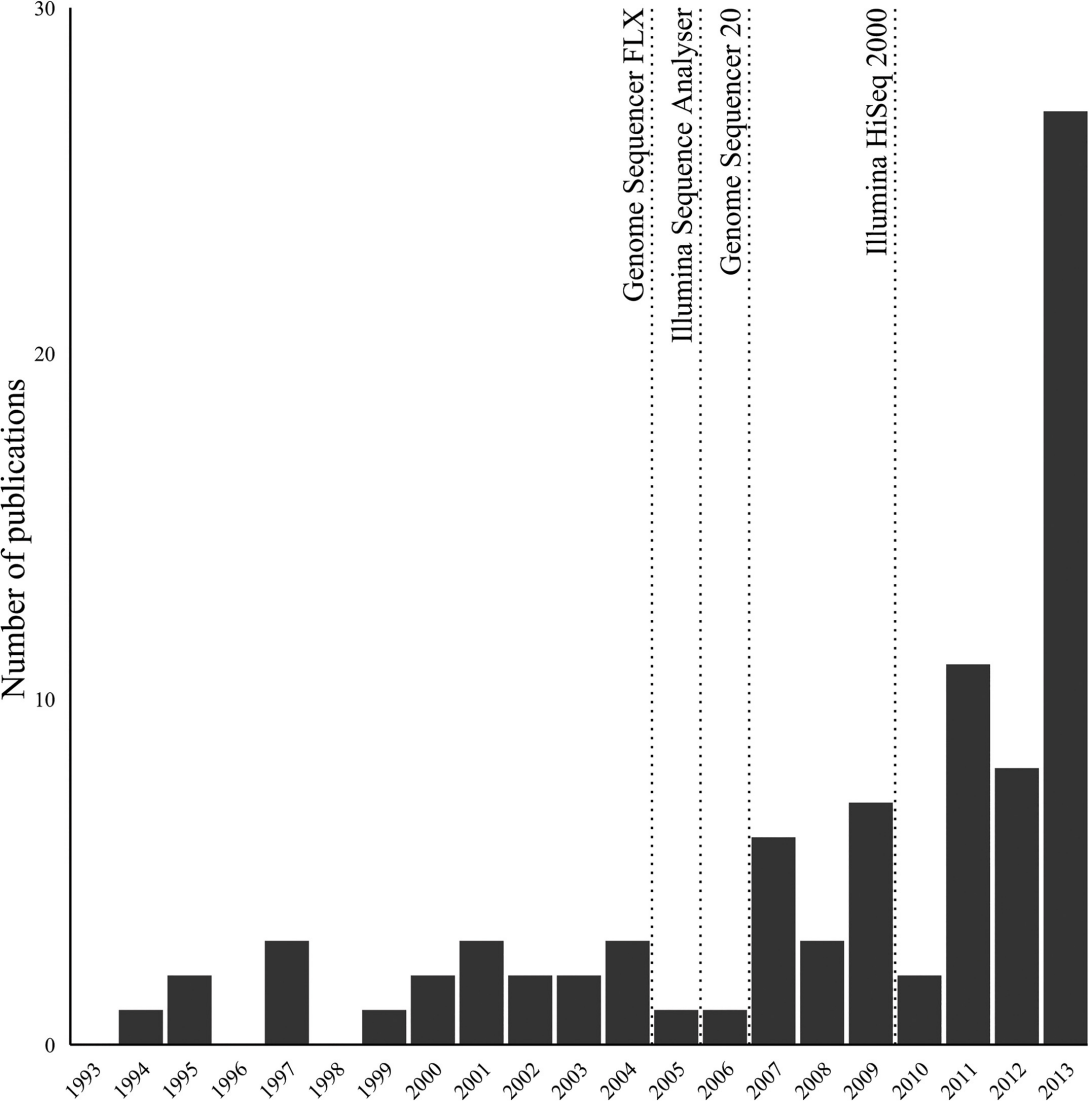
Reports of novel animal virus species in PubMed over the last two decades.

Next generation sequencing (NGS) platforms offer exceptional depth, speed and accuracy of sequencing, resulting in a significant increase in the rate of new pathogen sequences that have been identified from tissues or fluids [Fig 1]. The decline in sequencing costs [1] now makes this technology broadly accessible, with some recent viral discoveries having potential health and economic benefits [1–5]. NGS is also useful as an unbiased tool with the ability to identify previously undetected or unsuspected causative agents without prior information and has the potential to become a diagnostic tool overcoming inherent *a priori* limitations of conventional molecular diagnostics such as PCR and microarray technology.

Despite its success, the use of NGS for pathogen discovery is not straightforward. Any biological sample from a patient or animal tissue will inevitably consist predominantly of host-derived sequences. In some cases, the greater proportion of host genetic material will all but drown out any pathogen derived sequences leaving far too many single reads or assembled contigs to analyse.

Mapping subtraction to remove host reads from putative pathogen reads is often the first computational step used in many studies and pipelines [6–9] though the inverse also exists [10] by directly extracting putative pathogen reads by similarity to pathogen sequences in the public domain. These approaches use BLAST or optimal alignment algorithms against a range of publicly available data sets to, typically, identify sequence reads to subtract, thereby enriching the experimental data set for non-host (potentially pathogen derived) sequences prior to *de novo* assembly. However, reported validation/testing studies of this methodology are few or, where they do exist are limited, such as SURPI [9]. This direct comparison of alignment algorithms uses artificially generated human reads and 25,000 viral reads derived randomly from an NCBI ‘viridae’ search term and 3 NGS samples with known virus. The problem with this approach is firstly the viral database used (~1.2 million entries), which is heavily skewed towards a small number of viral species. Therefore taking a small subset of this is a) unlikely to reflect human viruses, b) unlikely to reflect molecular genetic breadth as defined by Baltimore viral classification and c) may be heavily skewed towards certain virus that are over-represented such as HIV-1. Secondly the human reads in this study are artificially generated and subtracted against a dataset that was the source of the artificial reads themselves resulting in an unrealistic scenario. Additionally, the existing pipelines cited above do not adequately examine potential downstream advantages of host read subtraction on assembly in relation to reduced contig numbers, changes to pathogen contig length and assembly accuracy.

*De novo* assembly of complete or subtraction-reduced read datasets into contigs can dramatically reduce the number of sequences that need to be put through homology search programs. The increased length of contigs over primary sequence reads allows greater certainty that homology hits are accurate. Additionally, when searching for novel pathogens, the generation of large contigs from sequence reads that do not possess significant similarity to any nucleic acid sequence in the reference databases may also indicate the presence of a novel, highly divergent pathogen. A potential problem with this method is the copy number of the viral pathogen itself. Despite the depth of sequence information from current NGS platforms, optimal coverage may be limited due to the sub-optimal quality of rare clinical samples or naturally low level of viral nucleic acids. For example, high quality total RNA from multiple biopsy sections of liver tissue naturally infected by Hepatitis C Virus (HCV), when Illumina sequenced, revealed only four viral reads per million [11]. The low coverage restricted the use of *de novo* assembly algorithms, a vital step in novel pathogen discovery. The use of *de novo* assembly is further complicated by the number of assembly programs available as well as the choice of settings to use within them. Unfortunately, direct comparisons of *de novo* assembly algorithms in the literature are often sub-optimally validated for these specific requirements or are unreported. More rigorous algorithm comparisons have been focused on bacterial genome characterization, many times larger and less variable than many viral genomes [12–20]. An assembly algorithm that is ideal for transcriptome assembly is often not suitable for genome assembly. Desai *et al* [21] have highlighted the need for improved testing of assembly algorithms emphasizing both the use of real data rather than a reliance on simulated sets and an increased focus on testing assembly accuracy rather than just contig length and number. A review of novel viral discovery literature and related pipelines [2, 3, 5, 9–10, 22–40] makes it clear that there is very little agreement between groups on the optimal viral discovery pipeline. More than half of the successful viral discovery papers reviewed used *de novo* assembly as a first step followed by a BLAST search of existing databases with no subtraction step. Additionally, assembly parameters varied from 100% similarity over 18 nt to 85% similarity over 25 nt and the BLASTn e-value parameter varied from 10 to 10–5. Others use a +ve or-ve subtraction process in the first instance. It is essential for this field to define optimal methodologies in order to circumvent both time and computationally inefficient tests but also to provide the researcher with high certainty that subtraction methodologies do not unnecessarily remove pathogen sequence just for the sake of speed and that available *de novo* assemblers are compared and optimized. Here we have used NGS data sets derived from naturally virally infected human liver biopsies, an artificial viral pathogen ‘metagenomics’ dataset modified using a profile based read emulator [41] in the context of a large human liver derived ‘host’ read dataset and idiopathic hepatitis liver samples, in order to explore and validate filtration, subtraction and assembly methods for novel viral discovery useful to the researcher directly or as an adjunct to existing fast pipelines.

## Results

Five Illumina Hi-Seq datasets derived from human liver tissue were used in this study, representing a cross-section of viral genome coverage and viral classes (see methods).

### Illumina sequence read host mapping subtraction and k-mer filtering

**Fig 2:**
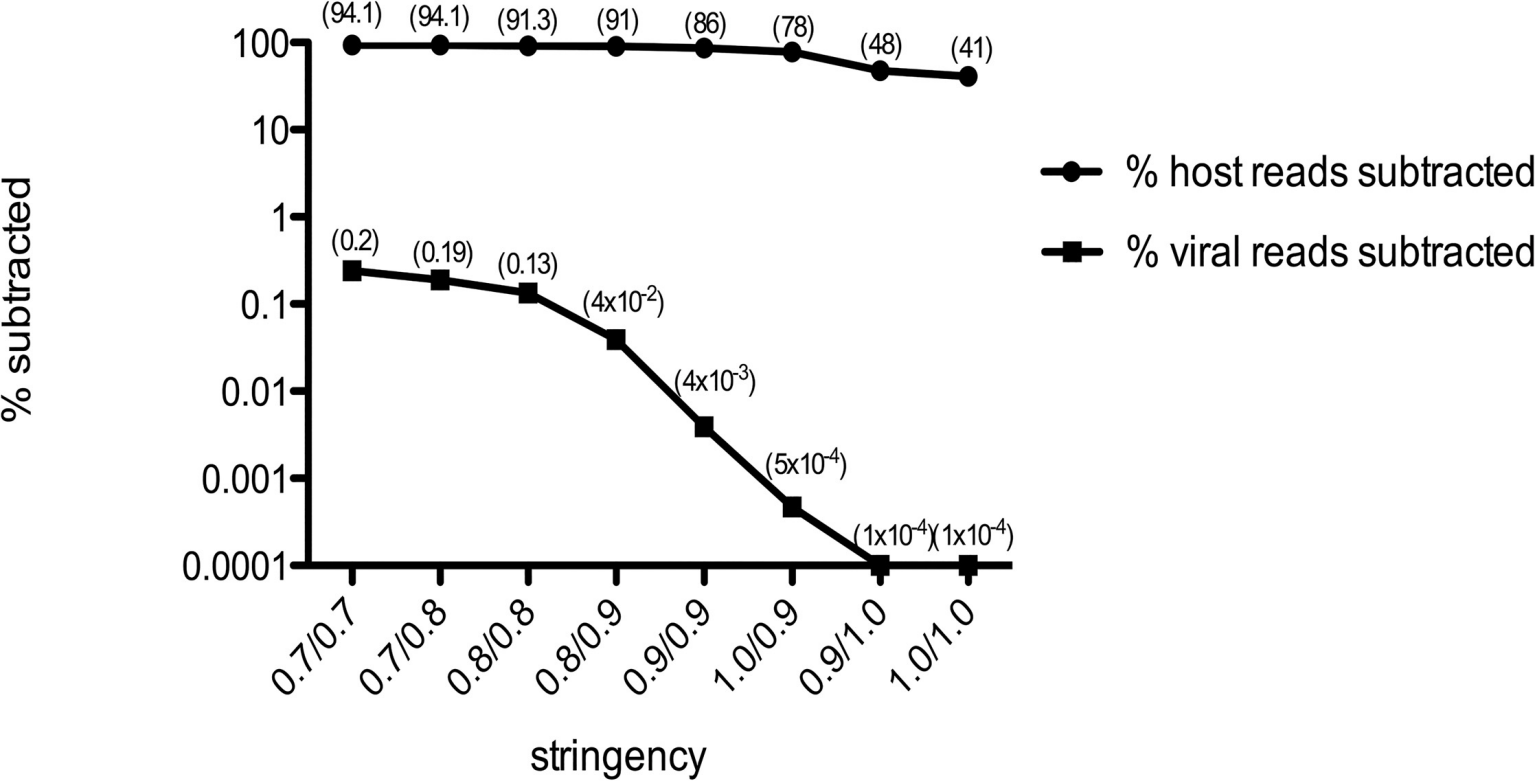
Illumina host / virus read subtraction by short read mapping algorithms (n/n = % of read / % identity).

**Fig 3:**
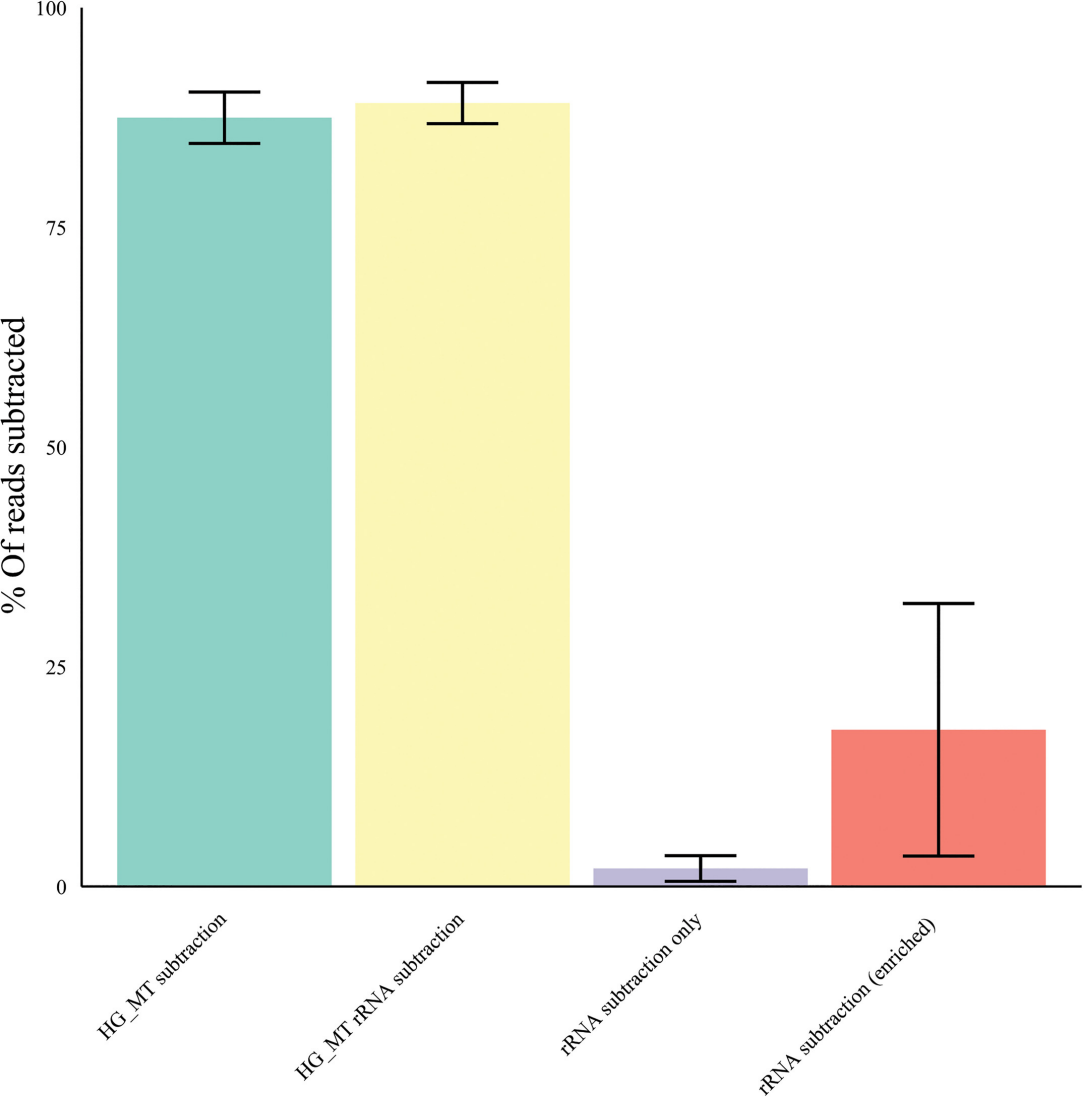
Host mapping subtraction by reference set.

**Fig 4:**
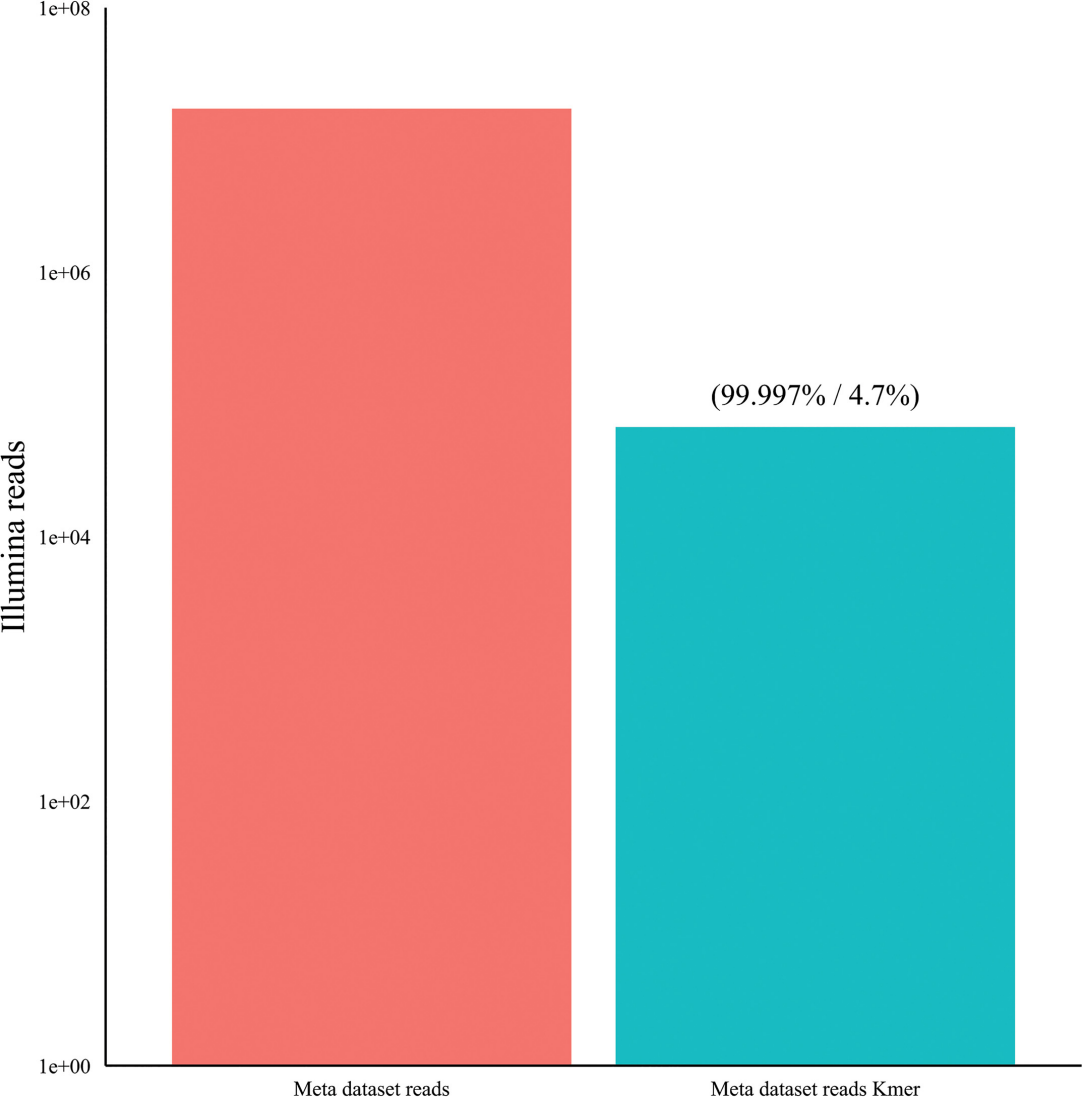
K-mer filtering of metagenomics Illumina read dataset (in brackets: % host sequence read subtraction and viral read subtraction respectively).

A database of 62 human virus chromatids from 35 distinct viruses were used to generate simulated Illumina read pairs (10x coverage depth) with the use of pIRS (methods). These paired reads (167,004) were combined with 16.7 million Illumina paired sequence reads from a ‘clean’ healthy liver sample (methods). Using the mapping algorithms CLC, BWA and Bowtie (methods) we investigated the percentage subtraction of host and viral sequence reads following mapping to human reference sequence data sets including the human genome, the human mitochondrial genome and a human rRNA sequence set (see methods) [Fig 2]. Mapper settings equivalent to 90% homology over 80% of the sequence read length (0.8/0.9) subtracted 95% +/- 0.2% of the host-derived Illumina reads without a major loss of viral sequence reads (0.02% +/- 0.005) for the algorithms tested [Fig 2] (Bowtie data not included for clarity). Analysis of the subtracted viral sequence reads at this mapping stringency revealed that they were exclusively homopolymeric tracts and / or repeats with a low % GC content. Consequently, this mapping stringency was used for subsequent validation studies. Mapping of the remaining (un-subtracted) sequences to the viral references revealed no gaps in coverage relative to the non-subtracted original read set. We further characterized the effect of subtracting host sequences using the human genome build/mitochondria and the rRNA reference sets independently, with Illumina (100nt, paired-end) data sets [Fig 3] derived from Total RNA, randomly primed and amplified, from idiopathic liver samples (n = 10). The greatest number of host sequence reads was subtracted through mapping to the human genome including the mitochondria (87.4 ± 4.3%, arithmetic mean ± standard deviation) with an average of 2.1 ± 1.3% of the host sequence reads subtracted by mapping to the human rRNA data set alone. The use of both reference data sets was additive, with 89.2% of Illumina reads removed. Additionally we applied hostmapping subtraction to our rRNA references with Illumina paired read sets (n = 10) derived from nuclease treated liver cytosolic samples (an encapsidated virus enrichment protocol) [8] (see enriched, Fig 3). Mapping to the rRNA set subtracted 17.9% ± 13.6% of the reads showing the potential benefit for rRNA mapping subtraction prior to assembly in this context. We next tested the effect of sequence read subtraction using Kontaminant (methods), a newly developed tool for read filtering developed at the Genome Analysis Centre (TGAC), Norwich, UK [42]. We again used our artificial Illumina paired–end viral read set embedded in healthy liver derived Illumina paired-end reads (as described above). Following k-mer filtering the data set was reduced to 67,432 reads. 99.997% of the human liver reads and 4.7% of the viral reads were removed (not including duplicates) [Fig 4]. The GC % content profile of the viral reads that were filtered mirrored the unfiltered viral reads. Additionally, mapping of the remaining (unfiltered) reads to the reference viral genomes revealed that minimal gaps in coverage were present (0.6 ± 0.6%) at the reference terminal ends only, relative to the unfiltered read set.

### *De novo* assembly comparison and optimal word size validation

**Fig 5:**
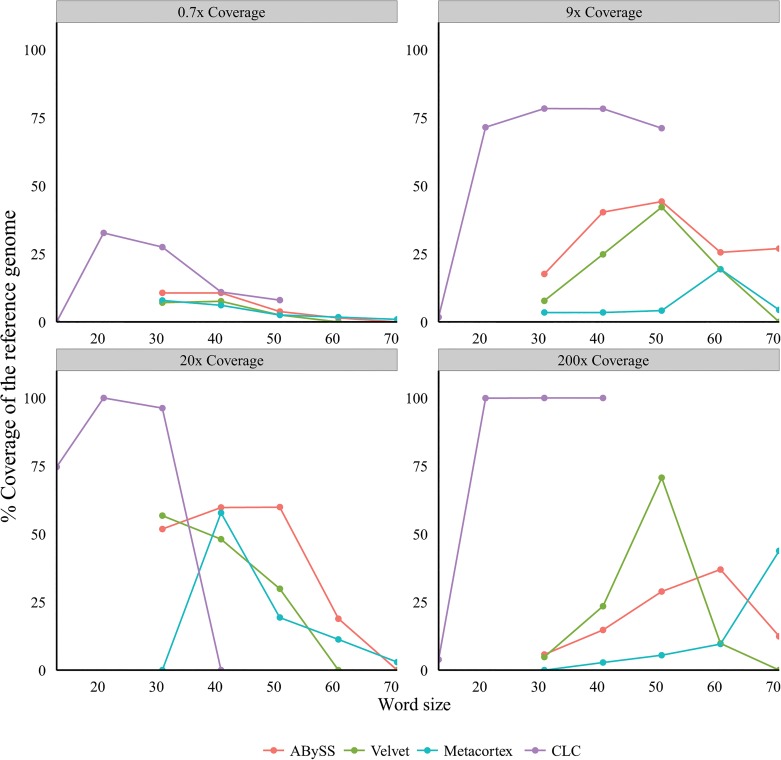
Optimal word size for viral assembly with multiple assemblers.

We compared a range of assembly algorithms and word size settings using four real Illumina datasets: two from HCV infected liver (containing viral reads with a mean reference coverage of 0.7 and 9x) and two from HBV infected liver (containing viral reads with a mean reference coverage of 20 and 200x). The reads were assembled using four algorithms: Velvet [43], MetaCortex [44–45], ABySS [46] and CLC and a range of word sizes. ABySS, Velvet and CLC are genomic assemblers, while MetaCortex is a development of the Cortex assembler aimed at highly diverse metagenomic datasets. The longest viral contigs assembled (as a % of the reference genome length) by each algorithm over a range of word sizes is shown in Fig 5. The CLC algorithm consistently produced the greatest length of reference genome coverage for every sample across the range of depths of viral sequence coverage. Interestingly, the assembly programs Velvet, MetaCortex and Abyss were generally less effective at the 200x deep viral coverage compared to the 20x coverage. The artificial viral sequence dataset was also *de novo* assembled using CLC v6.5 with extensive testing of the word sizes revealing an optimal word size of 21 (data not shown).

### Optimal word size assemblies following k-mer filtering and host-mapping subtraction

**Fig 6:**
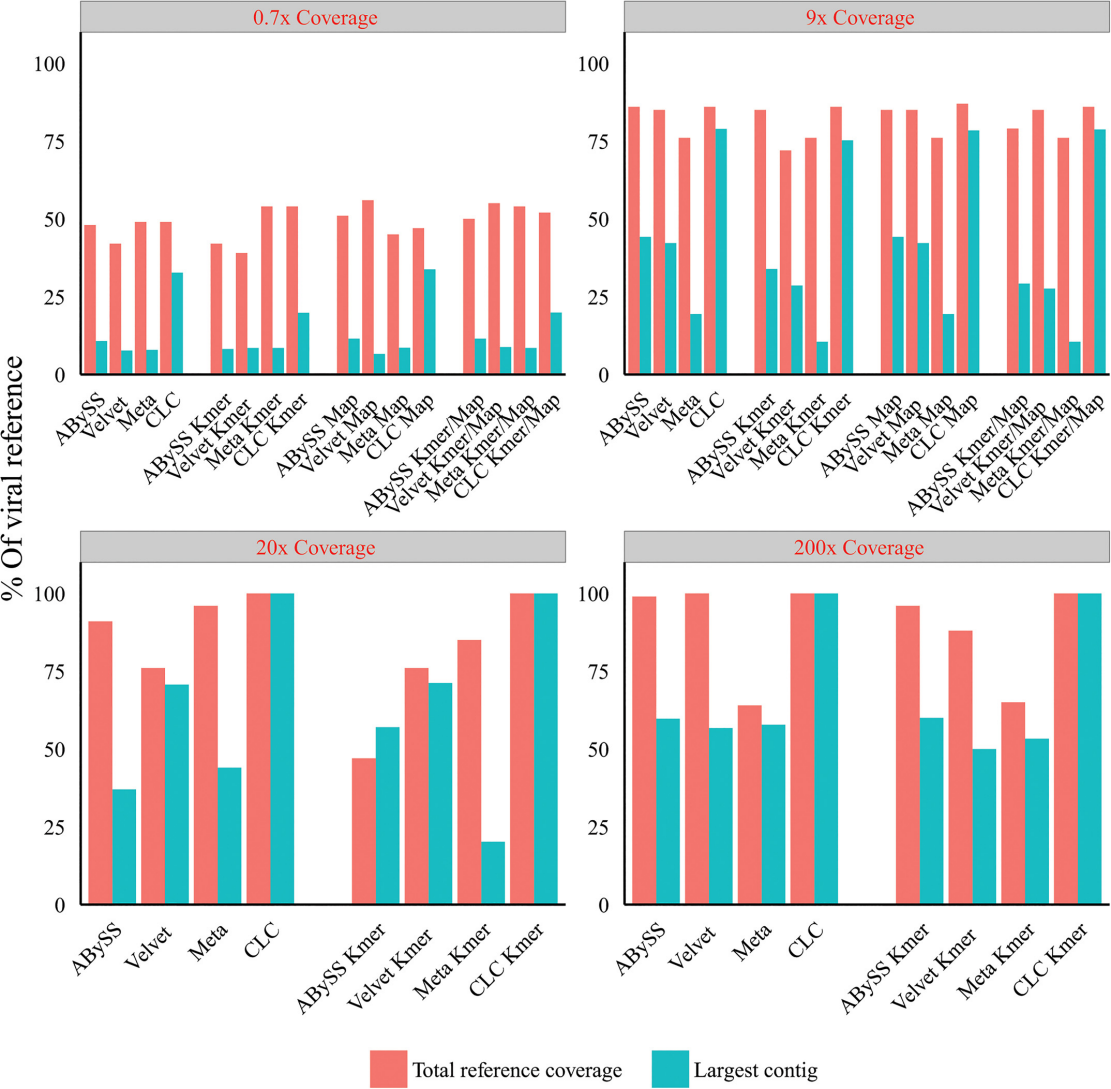
Effect of viral reference coverage of Illumina reads (red text), host mapping subtraction (Map) and k-mer filtering (K-mer) on viral contig size and reference coverage (post-assembly) using different assembly algorithms (Meta = metacortex). Each assembly algorithm used at optimal k-mer size.

With the optimal word size parameters for each assembly algorithm determined, we investigated the effect of host mapping subtraction and k-mer filtering on resulting *de novo* assembled viral contigs [Fig 6]. Pre-assembly k-mer filtering alone (across the range of assemblers and data sets) resulted in changes in the total reference coverage (of all assembled viral contigs) of -12.5% to +10.2% relative to assembled reads alone. The CLC and MetaCortex assemblers, post k-mer filtering, increased total reference coverage by +10.2% with Abyss and Velvet having a negative effect of -12.5% and—7.1% respectively. The largest single viral contigs as a percentage of the reference viral genomes across the range of assemblers and datasets was -23% to +10.4% relative to assembled reads alone, with the most pronounced negative effect observed with the lowest coverage set (0.7x depth of coverage). Pre-assembly host-mapping subtraction alone (0.7x and 9x deep viral coverage sets) resulted in changes to the total viral contig coverage of the reference across the range of -8.1% to +33.0% relative to the assembly alone. The range for the single largest viral contig size was -14.3% to +9.7% across the range of assemblers and datasets relative to assembly alone. This was skewed by the very negative result from the Velvet assembler without which the range was -0.9% to +9.7% with an average maximum contig size increase of 3.2%. Pre-assembly combination of host mapping subtraction and k-mer filtering on the 0.7 and 9x deep viral coverage sets. Total viral contig coverage of the reference and the single largest viral contig size was comparable to the k-mer only filtering with the exception of the 9x coverage Abyss data that showed a small reduction (4%) in the largest contig size relative to assembly alone.

### Effect of k-mer filtering and / or host mapping subtraction on the number of contigs assembled *de novo*

**Fig 7:**
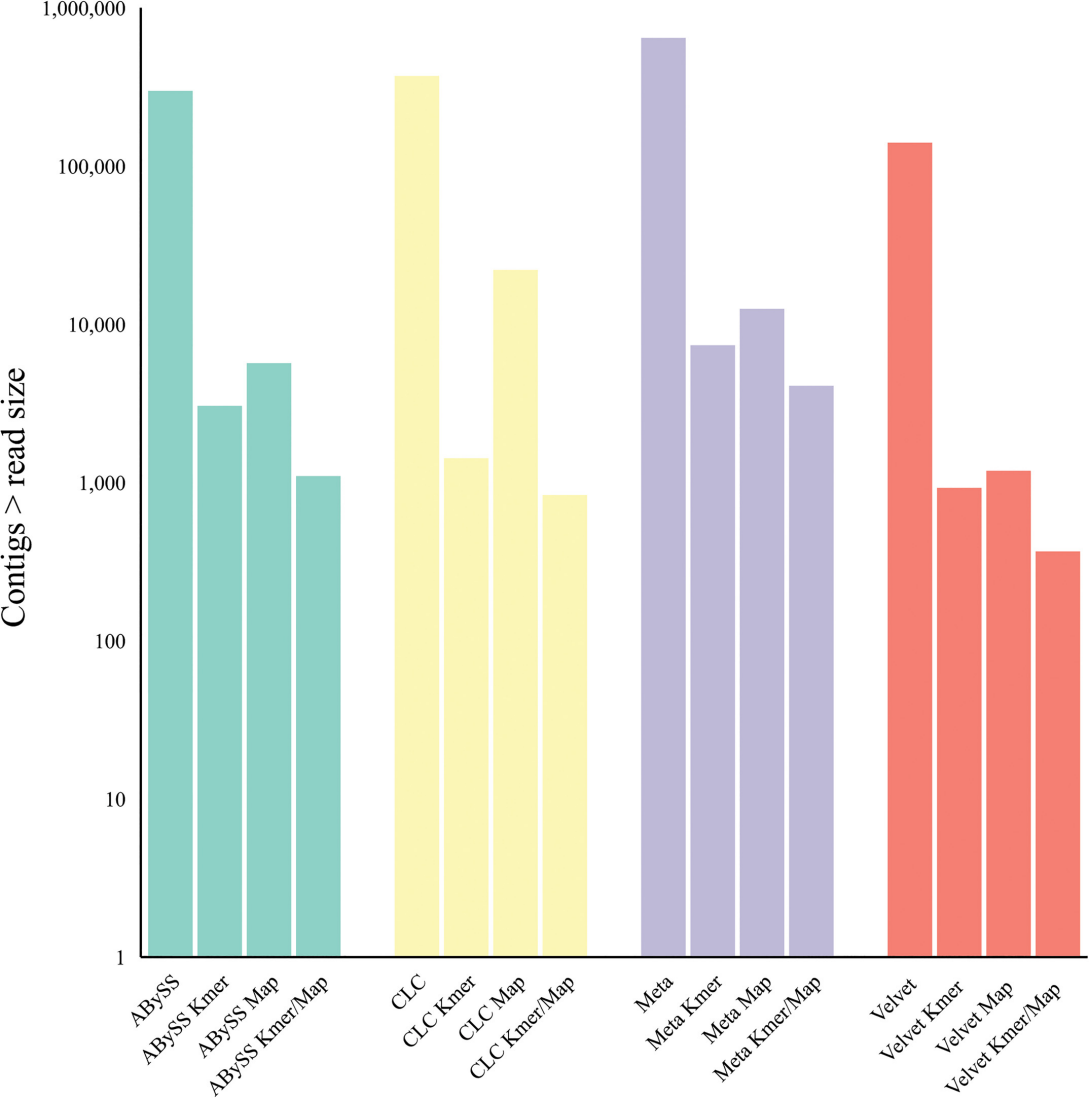
Effect of k-mer filtering (K-mer)/ mapper subtraction (Map) on post-assembly contig number using multiple optimized assemblers with the HCV 9x mean coverage Illumina read dataset.

Our primary objective was not only to determine and validate the optimal assembler algorithm, assembly parameters and the effects of read filtering methods but to assess whether the bulk read subtraction processes would consequently reduce the number of contigs assembled thus resulting in fewer contigs to analyse for putative virus. Assembled contigs less than, or equal to, the largest trimmed Illumina sequence read were discarded. For all assemblers tested, k-mer filtering reduced the number of contigs assembled by 98.7–99.6% and host-mapping subtraction reduced the number of contigs assembled by 98.5–99.6% [Fig 7]. There was an additive effect when combining both the k-mer filtering and host-mapping subtraction that reduced the number of assembled contigs by 99.3–99.8%.

#### DUST low complexity filtering analysis

**Fig 8:**
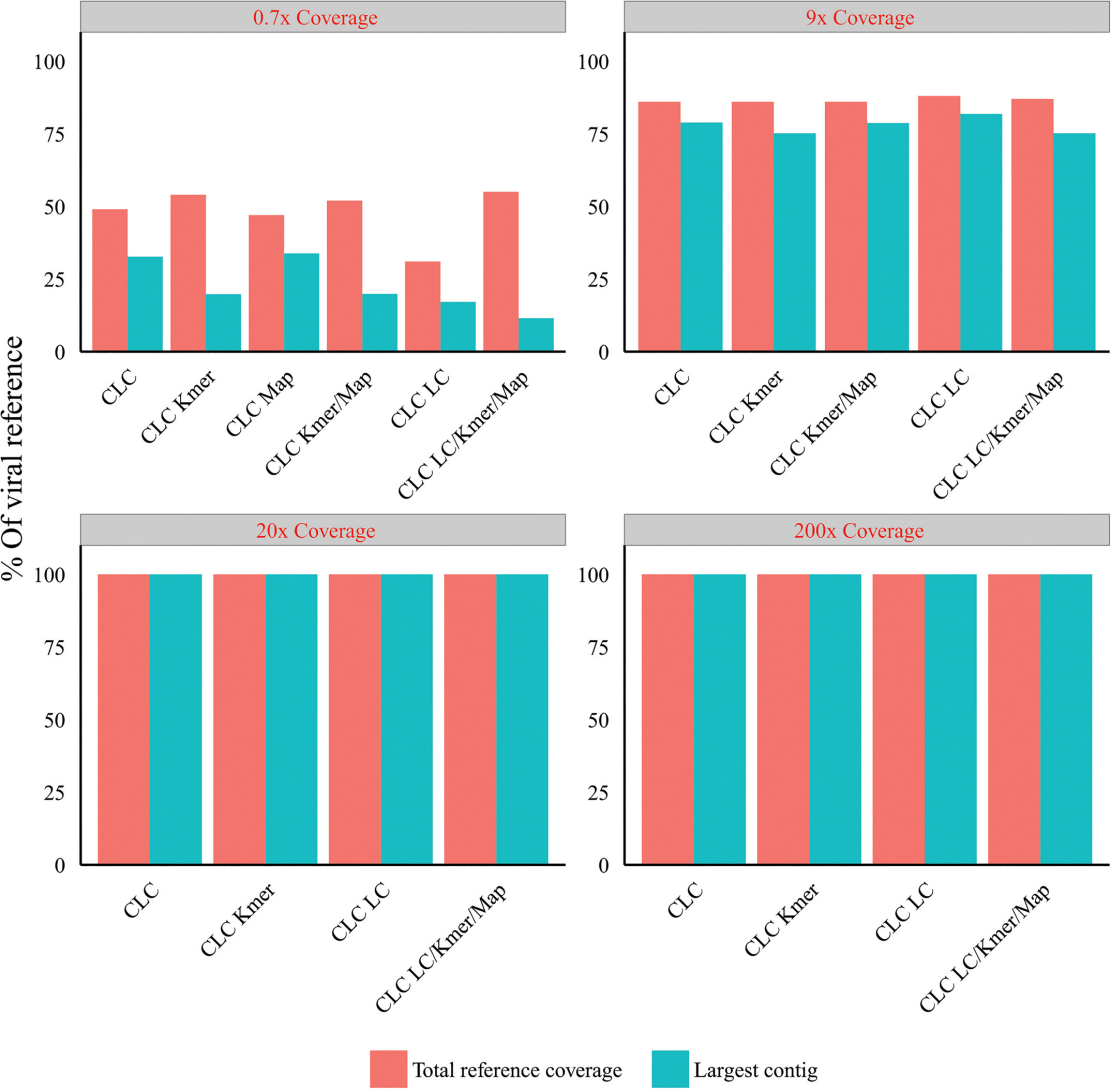
Effect of viral reference coverage of Illumina reads (red text), host mapping subtraction (Map), k-mer filtering (k-mer) and low-complexity filtering (LC) on viral contig size and reference coverage (post-assembly) using CLC assembler (v.6) at optimal word size.

**Fig 9:**
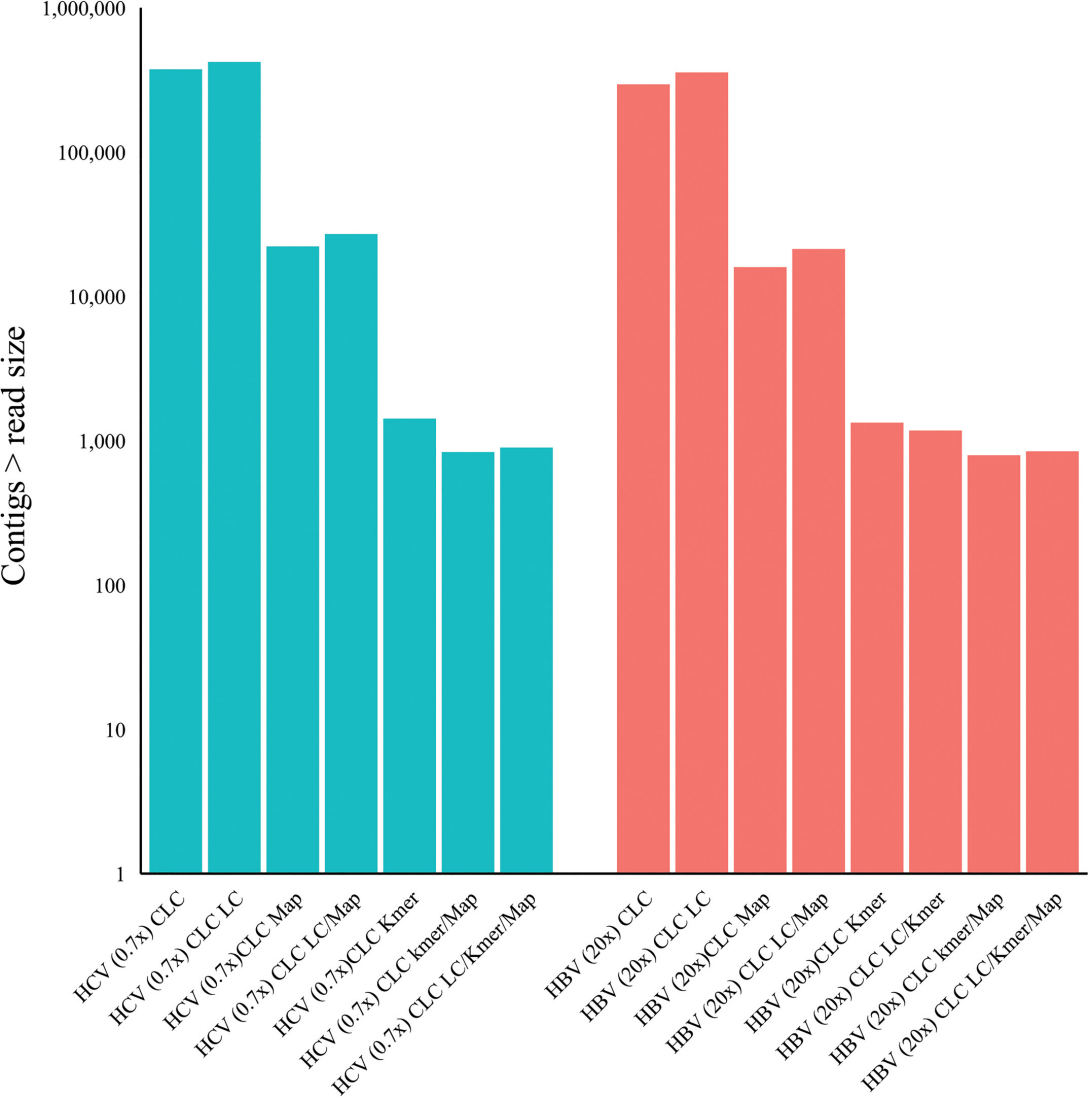
Effect of k-mer filtering (k-mer) / mapper subtraction (Map) and lowcomplexity filtering (LC) on post-assembly contig numbers.

We investigated the effect of filtering the NGS reads with a low complexity filter [47] (see methods) prior to assembly with the CLC assembler v6.5. We also investigated the effect of using DUST in combination with k-mer filtering and host mapping subtraction using the real Illumina NGS data sets with 0.7–200x depth of viral coverage. Filtering out the low complexity reads had no effect on the assembly of viral contigs, alone or in combination with other filtering/ subtractive processes for the 9x, 20x and 200x deep viral coverage sets [Fig 8]. However for the 0.7x viral coverage set, the use of the low complexity filter alone reduced the total length of the reference genome covered and largest viral contig size by 32% and 52% respectively compared to assembly with no low complexity filtering. A similarly negative effect was seen on the largest viral contig size generated when low complexity filtering was used in combination with host mapping subtraction relative to host mapping subtraction alone. Low complexity filtering together with k-mer filtering resulted in a similar output to k-mer filtering alone. The combination of host mapping subtraction, k-mer filtering and low complexity filtering negatively affected the largest viral contig assembled (a 47% reduction compared to k-mer filtering and hostmapping subtraction without the addition of low complexity filtering). Total reference coverage was comparable to assembly alone, the k-mer filtering and host mapping subtraction alone, or in combination. Overall, no advantage of using DUST was apparent. It is important to note that the 0.7x and 9x viral coverage read sets (both from HCV infected liver) did not include sequence reads spanning the 3’ proximal homopolymeric T tract of HCV. Thus the contig reduction observed was not due to removal of sequence reads containing this tract. We further investigated the effect of using DUST on the total number of contigs generated [Fig 9]. Overall the use of DUST, on its own or in combination with host-mapping subtraction, resulted in up to 33.8% more contigs assembled (greater than read size). However, a small reduction in the number of assembled contigs (<10%) was observed when DUST was used together with the k-mer filter relative to k-mer filtered sets without low complexity filtering.

### K-mer filtering and / or host mapping subtraction and resulting contig number reduction using an artificial viral ‘metagenomics’ dataset

**Fig 10:**
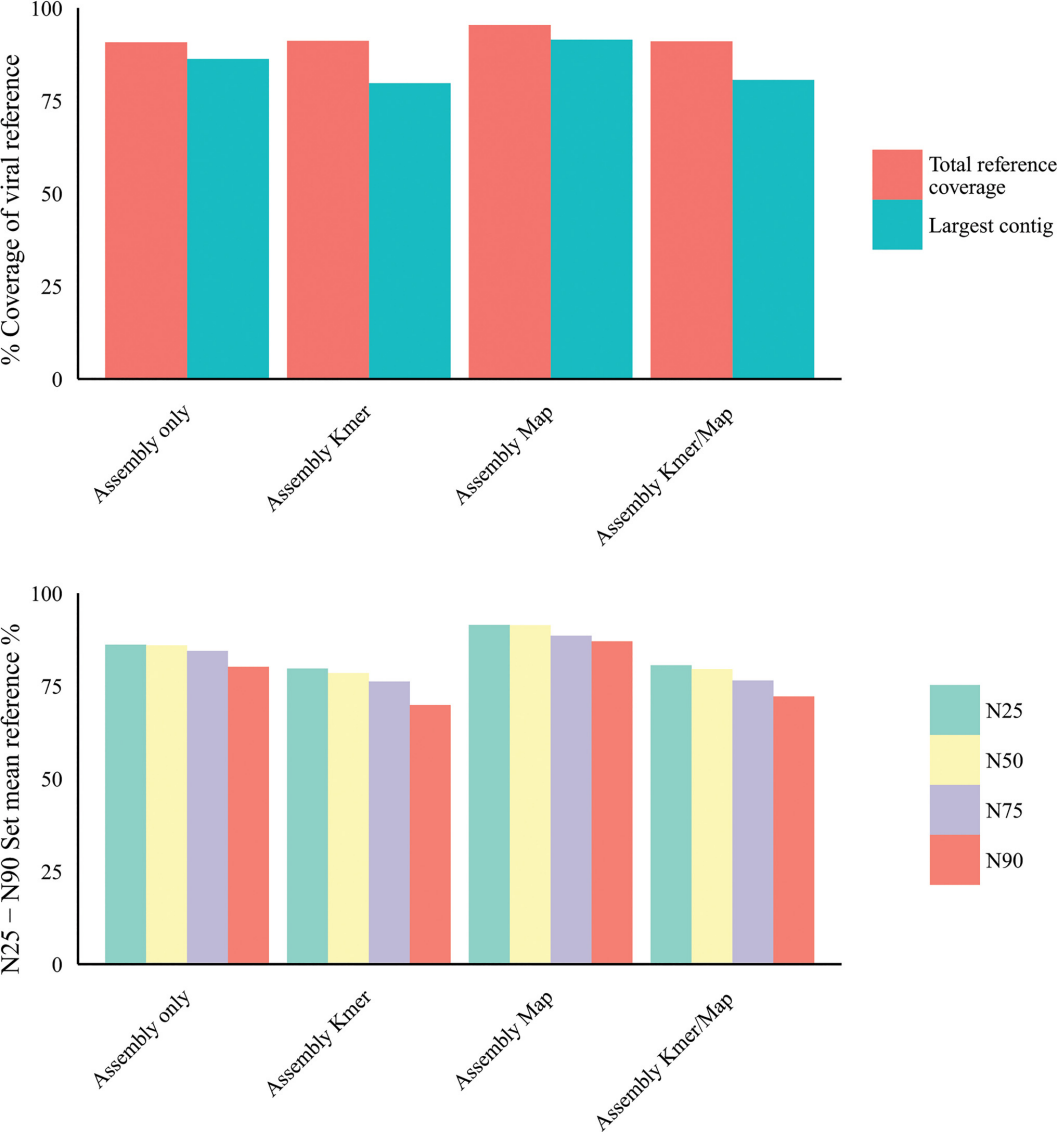
a) Simulated dataset: effect of k-mer filtering (K-mer) & host mapping subtraction (Map) on viral contig size and reference coverage. b) Simulated dataset: Effect of pre-assembly read filters on post-assembly N25-N90 (methods).

**Fig 11:**
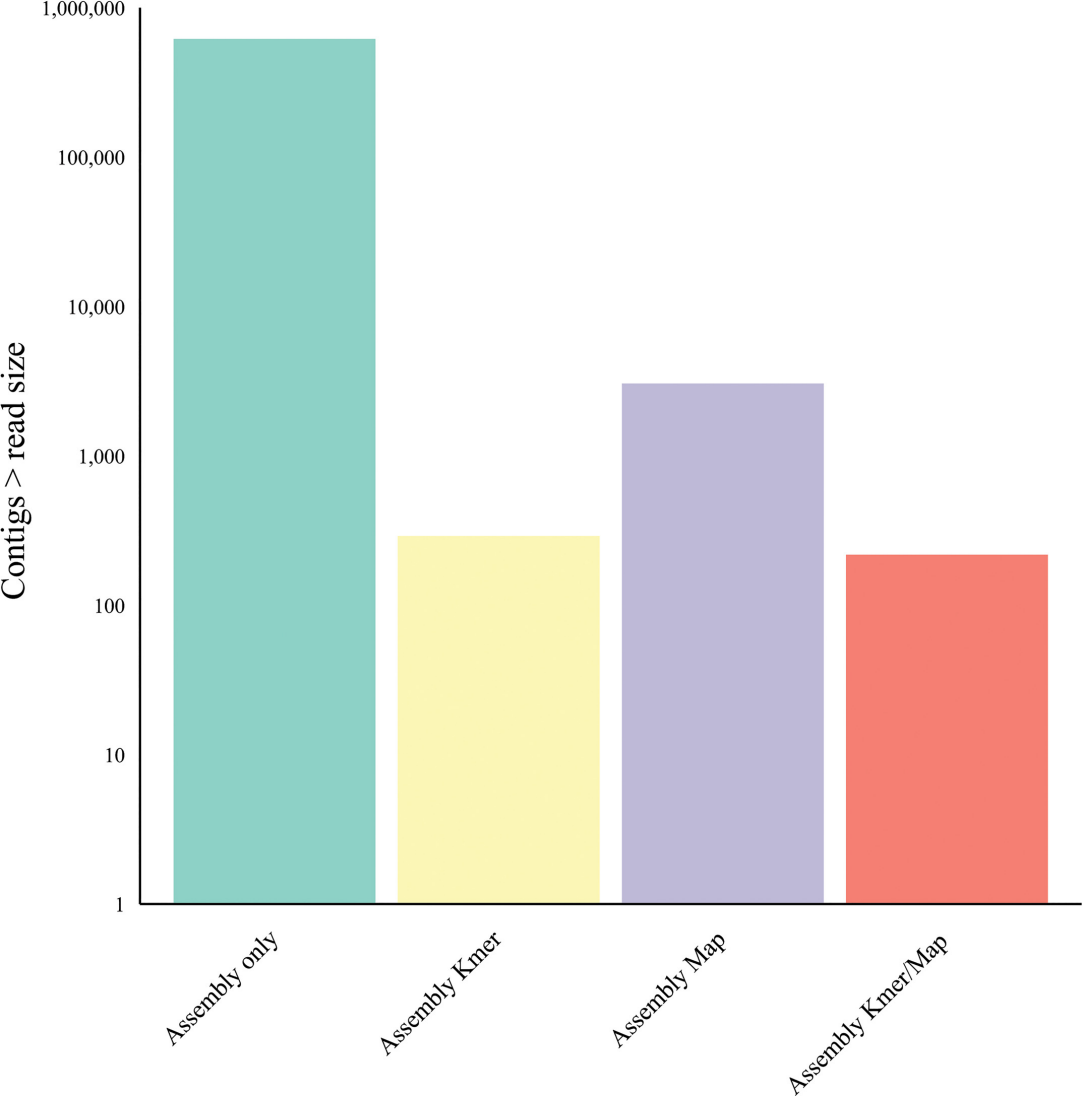
Human viral simulated dataset: effect of k-mer filtering (K-mer) & host mapping subtraction (Map) on post-assembly contig number.

To assess the effects of our pre-assembly filtering methodologies across a broader range of human viruses we used the artificial viral Illumina read set embedded in Illumina reads derived from healthy liver total RNA (described in methods). We applied the hostmapping subtraction and k-mer filtering (as before), prior to CLC v6.5 *de novo* assembly at optimal word size (as before). Contigs with greater than 90% homology to a viral reference genome were extracted from the assembled contigs and sorted by viral reference and percentage reference coverage. The mean reference coverage of the single largest contigs to each viral reference following assembly only was 86.3% ± 19.7% (arithmetic mean ± SD). Assembly following host-mapping subtraction only, resulted in mean reference coverage of the largest contigs of 91.4% ± 15.5%. Assembly following k-mer filtration only, resulted in mean reference coverage of the largest contigs of 79.7% ± 25.5%. Assembly following k-mer filtration and hostmapping subtraction together, resulted in mean reference coverage of the largest contigs of 80.6% ± 25.9% [Fig 10A]. N25-N90 values were derived for each reference (methods) and subsequently for each experimental group expressed as the arithmetic mean and standard deviation [Fig 10B]. Mean N90 values suggest large overlapping contigs without gaps as the smallest set mean value was 71% following k-mer filtering. Closer examination of the N25 and largest contig values for individual references revealed that in the control sample set (without read filtering or subtraction) the largest single contig range as a percentage of the reference was 52.7% to 99.7% with the exception of the herpes 1, herpes 8 and Vaccinia contigs at 15.2%, 26.1% and 35.4% respectively. For the host-mapping subtracted set, the largest single contig range as a percentage of the reference was 76.5% to 99.9% also with the exception of human herpesvirus 1, human herpesvirus 8 and Vaccinia virus contigs at 15.2%, 48.3% and 63.1% respectively. For the k-mer filtered set, the largest single contig range as a percentage of the reference was 32.1% to 99.8% again with the exception of the herpes 1, herpes 8 and Vaccinia contigs at 16.7%, 16.4% and 5.5% respectively. With respect to the total number of contigs assembled [Fig 11], 853,000 contigs were assembled without pre-assembly subtraction or filtering. Following host-mapping subtraction the number of contigs assembled was reduced to 4,700 contigs and with k-mer filtering alone 291 contigs were assembled. Applying both sequence subtraction methods prior to assembly reduced the assembled contig number further to 230, a total reduction in the number of contigs of 99.97%.

### Application of read subtraction filtering and optimal de novo assembly to idiopathic hepatitis liver samples

**Fig 12:**
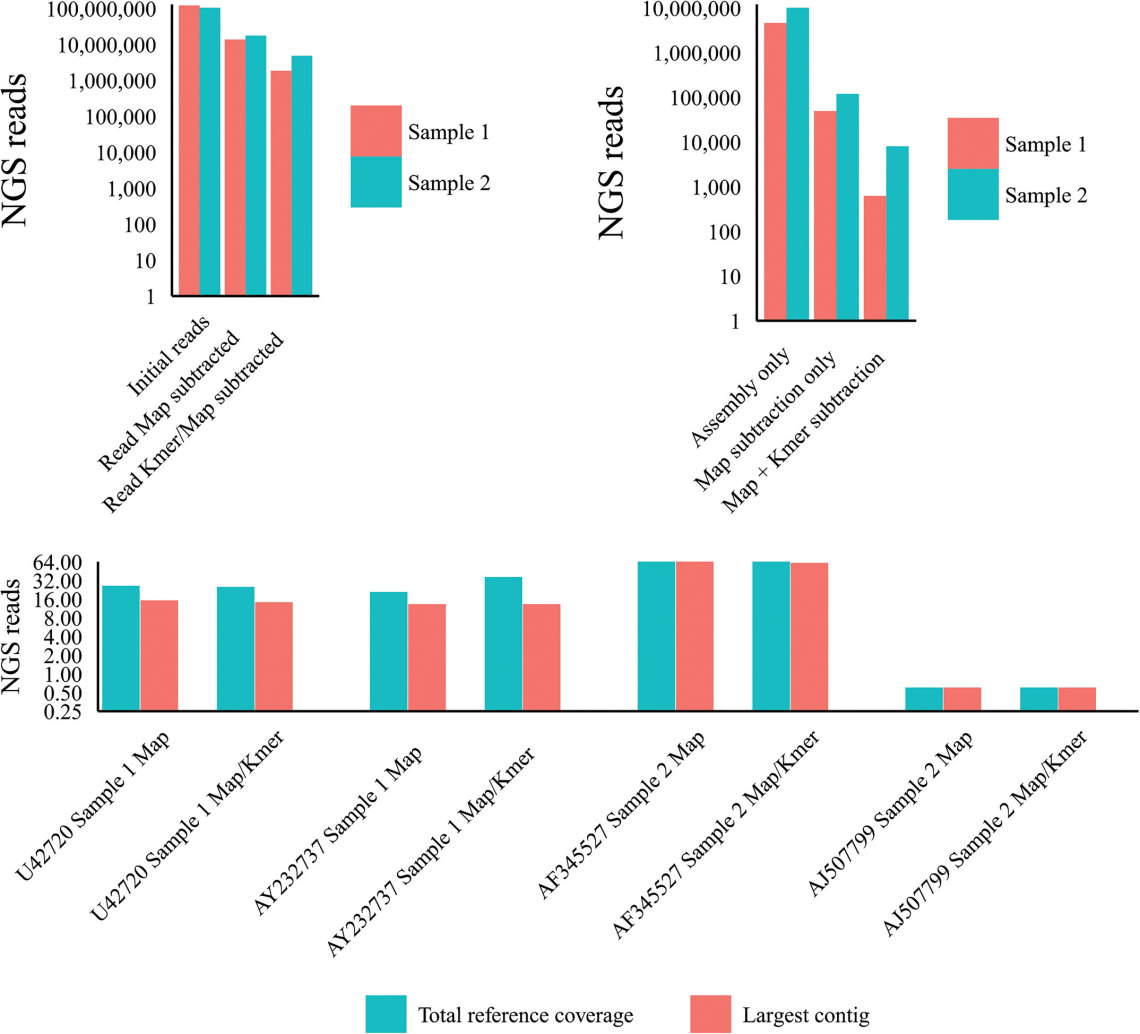
Idiopathic hepatitis liver datasets: a) Read reduction following mapping subtraction and k-mer similarity filtering. b) Effect of k-mer filtering (K-mer) & host mapping subtraction (Map) on post-assembly contig number. c) Effect of k-mer filtering (K-mer) & host mapping subtraction (Map) on viral contig size and reference coverage.

Two human hepatitis liver samples clinically defined as idiopathic (explanted liver from the transplant setting) and confirmed as negative for hepatitis associated viruses prior to transplant were processed for total RNA and SISPA processed for Illumina NGS as described in materials and methods. Sample 1 (121 million 100nt reads) and sample 2 (105 million reads 100nt reads) were filtered by the application of a short read mapper, with and without the application of the k-mer filter (Kontaminant). Read subtraction following these processes is shown in Fig 12A with subtraction levels comparable to the artificial metagenomics dataset and the control viral sets used previously. Optimal *de novo* assembly yielded contig numbers again commensurate with the previously tested control samples with an approximate decrease in contigs following the application of the short read mapper subtraction of ~100-fold for both idiopathic samples tested. Secondary application of the k-mer filter decreased the contig numbers further for samples 1 and 2 by 90% (620 contigs assembled) and 83% respectively (8027 contigs assembled) and shown in Fig 12B. These contig numbers are small enough to use BLASTn to NCBIntDB in a matter of hours and by tBLASTx in a few days using a standard 8-core desktop computer. To determine if the idiopathic samples included viral contigs we first blasted by both BLASTn and tBLASTx to NCBIntDB (materials and methods). For the purposes of comparison between pre-assembly filtering methods we considered NCBInt BLAST hits to be viral only following the application of high stringency cut-offs (as detailed in materials and methods). Best viral hit references were used to align the putative viral contigs from the different samples to ascertain the changes in the largest viral contig present and the total reference coverage of the viral contigs [Fig 12C.]. Between the two idiopathic samples, four distinct viral hits were detected, a) a retrovirus–like sequence with greatest homology to the SIV sequence U42720 complete cds (9068nt), b) HCV hits with greatest homology to the HCV2b sequence AY232737 complete cds (9711nt), c) TTV hits with greatest homology to the TTV sequence AF345527 complete cds (3208nt), d) a herpes-like hit with greatest homology to the HHV4 sequence AJ507799 complete genome (171823nt). The U42720 largest contig and total coverage were reduced slightly (12% and 7% respectively) by the addition of the k-mer filter pre-assembly. The AY232737 largest contig was reduced (1.3%) by the addition of the k-mer filter pre-assembly and total coverage was increased (82%) by the addition of the k-mer filter pre-assembly. The AF345527 largest contig was reduced (19%) by the addition of the k-mer filter pre-assembly and total coverage was increased (20.1%) by the addition of the k-mer filter pre-assembly. The AJ507799 largest contig and total coverage were unchanged by the addition of the k-mer filter pre-assembly. Overall, post-assembly data is consistent with our viral control and artificial datasets.

### Comparison with SURPI pipeline output

Using the SURPI pipeline in comprehensive mode [9] we ran three of our sample sets. Firstly, the 10x coverage metagenomics artificial viral set followed by the 2 low coverage real HCV datasets. All viruses were identified by SURPI. However, viral reads in our datasets are all strongly represented in the public domain and as such it is expected that all would be identified at the read level. We then analysed the viral reads that were removed at the pre-processing step, inappropriately removed by SNAP alignment to the human DB and finally we looked at the assembled contigs set and both the size of the largest viral contig and the total viral reference coverage of all the assembled contigs in order to directly compare the output to our processes.

For the two HCV datasets, viral reads were subtracted at the pre-processing step but not at the alignment to human DB step with a total subtraction of HCV reads of 4% and 5.3% for the 9x and 0.7x coverage HCV datasets respectively. This compares to 0% subtraction of reads using our trimming and human read short read alignment process as described. For the Artificial metagenomics viral dataset, SURPI pre-processing removed 0.43% of the viral reads and no further reads were subtracted by SNAP to human DB. This compares to 0.2% subtraction using our trimming and human read short read alignment process as described.

*De novo* assembled contig numbers were comparable between SURPI and our short read mapping subtraction followed by assembly for the three sets tested: Artificial/HCV 9x coverage/ HCV 0.7x coverage. For SURPI the contig numbers were 7563 / 42900 / 41957 respectively and for our described processes the assembled contig numbers were ~ 4700 / 26700 / 37400.

**Fig 13:**
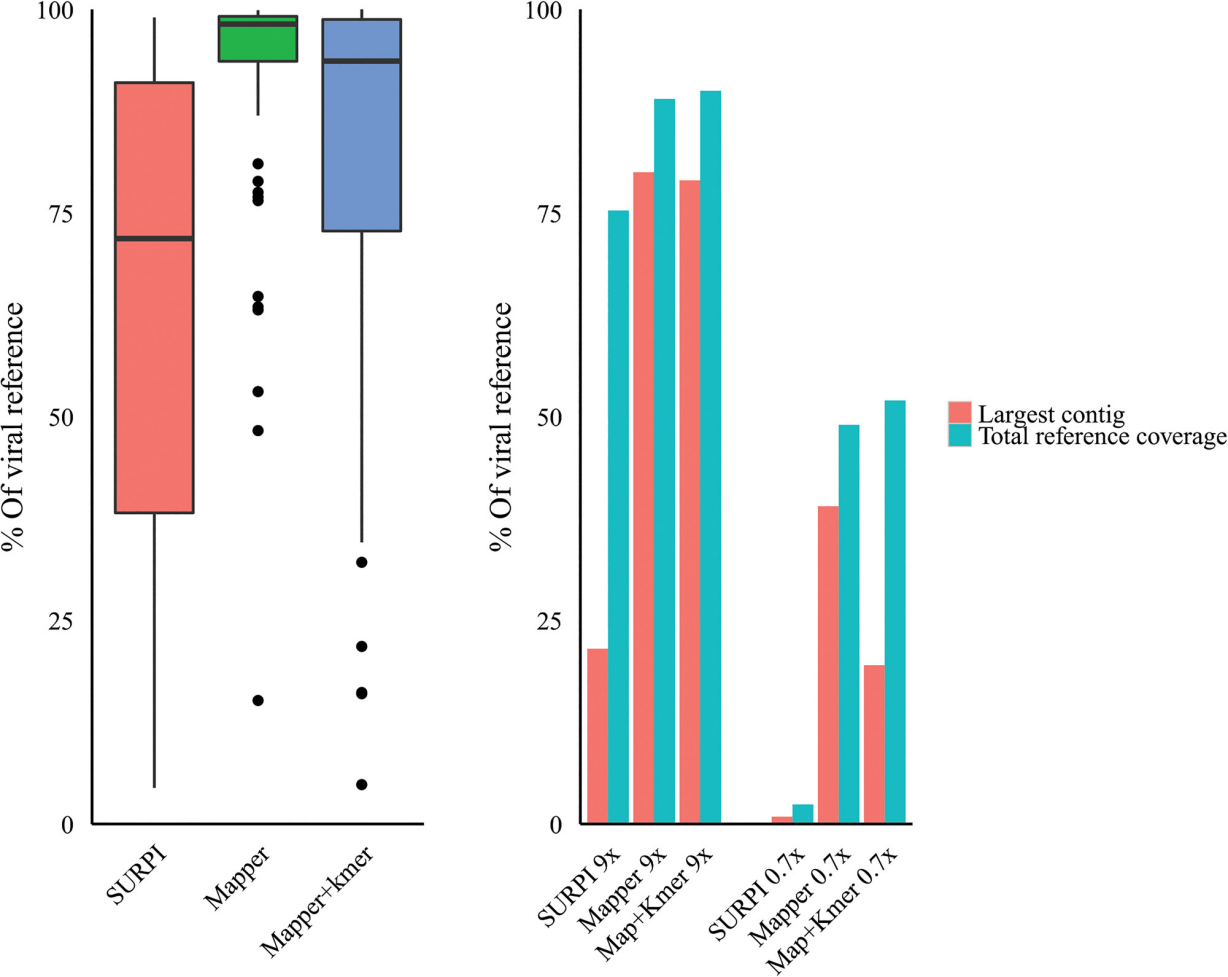
SURPI assembled contigs comparison: a) contig coverage of viral references (artificial metagenomics viral dataset) range and mean. SURPI SD = 28, Mapper SD = 15.5, MAP+k-mer SD = 25.9. b) HCV viral infected liver tissue NGS datasets at 9x and 0.7x coverage with Largest viral assembled contig (blue) and total viral reference coverage of all contigs (red).

The SURPI pipeline uses ABySS + Minimo to assemble reads negatively selected by SNAP to pathogens together with viral SNAP aligned reads. The assembled contigs generated were aligned by us to the reference sequences to ascertain the largest contigs and the total reference coverage of all the assembled contigs and compared to contigs generated by our processes as described [Fig 13].

Our mapping and assembly process using the artificial viral metagenomics dataset [Fig 13A] shows a larger contig size mean (91.4%) and higher lower range (15.2%) with an SD of 15.5, compared to SURPI with a mean of 65% of the viral reference and a lower range of 4% and an SD of 28. The additional use of the k-mer filter tool results in a still superior 80.6% mean and a lower range of 5.5% with an SD of 25.9. Additionally, the dramatically reduced contig numbers (k-mer filtered) following assembly can be readily used to extract the larger contigs.

Analysis of our HCV sets allows some consideration of low (9x) coverage and very low (0.7x) coverage with datasets derived from naturally virally infected human tissue [Fig 13B]. For the 9x coverage sets, SURPI contig assembly yielded a largest contig of 22% and total reference coverage of 76% for all contigs. This compares to 80% and 89% respectively for our short-read subtraction and assembly process without k-mer filter applied pre-assembly. The additional use of the k-mer filter had little effect at 79% and 91% respectively. Analysis of contigs generated from the very low coverage (0.7%) dataset, the SURPI assembled largest contig was 2% of the reference and total reference coverage of all viral contigs assembled was 3.5%. This compares to 37% and 46% for our processes without k-mer filtering and with the addition of k-mer filtering (pre-assembly) the coverages were 19% and 53% respectively.

## Discussion

The identification of viral sequences by NGS, in eukaryotic cells and tissues is problematic despite the development of enrichment methodologies [11, 48]. Problems include homology of viral sequences to host, the low ratio of virus to host sequence reads, and the low absolute number of viral reads which restricts the use of assembly algorithms to generate larger viral contigs. Perhaps most importantly, this field lacks adequately validated computational methods for subtraction of non-viral (host) sequence reads from NGS data sets whereby the ‘filtering cost’ (the chance of losing viral sequences following the application of host sequence filtering methods and the effect on assembled contigs) is ascertained and reported and where multiple algorithms are legitimately compared with real as well as synthetic sample sequence sets. Additionally, the large number of contigs generated from *de novo* assembly of unfiltered data sets restricts downstream analysis. We have explored and validated a range of computational filtration / subtraction methods using a combination of Illumina NGS data sets derived from viral infected liver tissues covering a range of viral coverage depths, together with an Illumina read simulated data set containing a broad range of viral sequence reads embedded with a large dataset of non-synthetic human liver reads. In the context of viral discovery, we have characterized the effects of these pre-assembly methods according to changes in post-assembly viral contig size, viral genome coverage of all contigs and the total numbers of contigs assembled (a potentially important factor with down-stream contig characterization) as well as back to back comparisons of several popular *de novo* assemblers.

We first characterized NGS read reduction potential using two distinct approaches [Figs 2–4] together with optimal k-mer word size optimization for a range of assembly algorithms [Fig 5]. Defined host-mapping subtraction, k-mer filtering and *de novo* assembly settings were then applied to our five data sets [Figs 6–11] and two idiopathic hepatitis liver datasets [Fig 12]. Post-assembly analysis of our data suggests host read mapping subtraction using a short read mapping tool did not compromise, in some cases significantly improved, the likelihood of assembling longer viral contigs. Furthermore, we have shown that this subtraction method will dramatically reduce the number of contigs subsequently assembled. Unsurprisingly, given our efforts to standardize the mapping algorithms we compared, we did not see a great difference between them. In terms of bulk reduction of host sequence, the removal of viral reads and the effect on assembled contig numbers, these algorithms are largely similar and ultimately it is up to the user to choose the stringency according to their own cost / benefit assessment. Nevertheless, we have shown that parsing the unmapped reads according to differing percentage read length and similarity mappings will allow the subtraction of the vast majority of host reads with very little likely loss of viral pathogen sequence. However, the point at which viral sequences begin to be removed by these short read aligners still leaves a large number of real host reads unsubtracted from our test dataset. The consequence of this is that the number of contigs assembled by subsequent *de novo* assembly is still very high and remains computationally expensive and time consuming to screen by homology to a complete nucleotides database particularly at the amino acid level. To attempt to further subtract the host reads with a view to further reducing the number of contigs subsequently assembled we employed the contaminant removal software (Kontaminant). Host read subtraction using Kontaminant was greater than subtraction using the short read mapping tools tested but with the consequence that, post-assembly, the maximum viral contig sizes as a percentage of the viral references were generally reduced relative to identical sets that were not filtered using Kontaminant. However, considering all five of our read sets together, the increase/ reduction in largest viral contig size, following the application of Kontaminant (pre-assembly) ranged from a 66.7% reduction up to an increase of 30.4%. The overall mean showed a reduction of 6.53% with an SD of 20.59%. We believe that this negative effect would, in general, not compromise viral characterization particularly when viewed in its proper context, as an initial first-pass approach to viral discovery in short read NGS data derived from eukaryotic tissues. In this context, the primary requisites are confidence that the assembler chosen and the k-mer size choice is appropriate to maximize viral contig size and that the total number of assembled contigs is reduced without removing putative pathogen sequence, facilitating the speed and ease of post-assembly analysis. The use of Kontaminant might therefore be considered as a 2nd step process after the optimized first step use of short read aligners (BWA, BT2 CLC mapper etc). The further reduced (post-assembly) contig set from step 2 can then be used to extract the longer, more complete contigs assembled after step 1 only.

We found no clear advantage in the application of a low-complexity filter (DUST) to our Illumina read sets pre-assembly, alone or in combination [Figs 8–9] which may be due to the read trimming we have applied. Any negative effects seen with the dust module may be due to the removal of viral reads suggesting a lower stringency could be applied but given the largely neutral effect it is unlikely that this would then offer an additive benefit to the short read mapping subtraction strategy.

The use of the PIRS software will not introduce the same level of sequence bias that may be seen with randomly amplified nucleic acids and this potentially remains an issue with the use of artificial datasets. However, the application of the optimized mapping subtraction protocol and k-mer filter (Kontaminant) to our idiopathic hepatitis liver samples (total RNA processed for NGS at very high depth greater than 100 million reads) shows post-assembly data that is consistent with our viral control and artificial datasets [Fig 12]. Comparison with the SURPI pipeline [Fig 13] indicates that SNAP is a good choice as a human database subtraction aligner with assembled contig numbers following this process broadly equivalent to our 1^st^ step processes. Viral reads lost at the SURPI pre-processing step may be due to the inclusion of the dust module and may be relatively more important were viral coverage is very low. The poor performance of the de-novo assembly component of the SURPI pipeline again highlights the need for rigorous optimization to increase viral contig size if one is to maximize the potential for identifying highly divergent viral species rather than ultimately relying on high identity with viral sequences in the public domain with short Illumina reads.

Taken together, the commonly encountered difficulty of analyzing many thousands of *de novo* assembled contigs in a search for putative viral sequence from Illumina NGS data can be made more manageable by removing the majority of host nucleic acid derived sequences prior to assembly by the application of mapping tool subtraction sequences and host k-mer frequency based filtering to host references, separately in a two step process. The first step consists of subtraction using standard aligning tools (BWA, BT2, CLC mapper etc.) which can be validated using a broad range of viral sequences modified by an emulator to mirror genuine host read data in which they are embedded. Stringencies defined to maximize the subtraction of host whilst minimizing subtraction of viral sequences can then be applied to the novel sequence datasets to provide a reduced set that significantly improves the subsequent assembly of viral contigs whilst ensuring that the assembled contig number is dramatically reduced for subsequent direct analyses if required. Step 2 entails the use of a k-mer frequency filtering system (Kontaminant) in order to remove most of the remaining host reads with the effect that the post-assembly contig number is further reduced whilst likely retaining all representatives of the viral contigs which can be used to extract the larger assembled contigs from step one. The overall dramatically reduced set is small enough to directly use BLASTn and even tBLASTx to a complete NCBInt database due to the contig numbers typically being less than 10^3^ (+/-50%) determined by us using over 50x idiopathic liver sample (total RNA) Illumina sequenced with a read depth average of 160 million placing this ‘high certainty’ viral discovery approach in the hands of the small laboratory with standard desktop equipment.

We compared three *de novo* assembly algorithms and a single specific metagenomics assembler, demonstrating striking differences between them and highlighting the need to compare and optimise the use of assembly algorithms in the context of viral discovery from human tissue derived NGS datasets. In theory, metagenomic assembly tools should be well suited to low coverage pathogen sequences in the context of tissue derived host sequences and many additional assemblers are worth comparing as has been recently undertaken together with an ensemble strategy [49].

## Methods

### Ethics Statement

Human liver samples were acquired from the Institute of Liver Studies, Kings College Hospital, London, University of London, UK. Samples were obtained with patient written consent. This work forms part of a broader project with ethical approval provided by the UK National Research Ethics Service, Cambridge 3 Research Ethics Committee, Cambridge CB21 5XB (REC reference numbers 09/HO306/52, 09/HO306/60) and Kings College Hospital Research Ethics Committee, London SE5 9RS (REC reference number 04/Q0703/27).

### Tissue samples and derived datasets

Two liver samples naturally infected with HCV and HBV viruses together with an uninfected ‘healthy’ liver sample were used in this study. Total extracted RNA and cytosolic viral particle enriched fractions (for each sample) were prepared using the SISPA protocol and sequenced using the Illumina platform (GAII for the HCV and HBV controls and Hi-Seq 2500 for all other samples).

The five controlled test sets used in this study have been previously reported [11]. They include two HCV infected liver datasets with 0.7x and 9x mean depth of viral genome coverage and two HBV infected liver datasets with 20x and 200x mean depth of viral genome coverage in addition to a uninfected healthy liver dataset. Additionally, two liver samples from explanted livers from the transplant setting (clinically defined as idiopathic and negative for known hepatitis causing viral infections) were also used [Fig 12]. Illumina Datasets are available from http://www.ncbi.nlm.nih.gov/sra/ with the accession numbers ERX180664, ERX180665, ERX180666, ERX180667, ERX286289.

### Illumina sequence read trimming and quality control

Prior to sequence assembly and / or host sequence read removal, we trimmed all Illumina paired read data sets using CLC Bio v5.5 Trimmer (Aarhus, Denmark). SISPA PCR primers used for random priming and amplification [11] were removed (+/- strand search setting). Other settings chosen included: max ambiguity/read = 3, reads discarded if <38nucleotides in length (following all other trim functions). Quality control analysis revealed sequence read level (arithmetic mean) PHRED score averages of over 40 for all sets, with no single read mean PHRED score less than 30.

### De novo assembly algorithms

The HCV and HBV sets (0.7x–200x viral coverage) were used in the first instance to ascertain optimal word size values with four assembler algorithms. Velvet 1.1.04 [43], MetaCortex 0.1 [44–45], ABySS 1.3.4 [46] and CLC Bio v6.5 (Arhus, Denmark). K-mer size was varied from 21 to 71 in steps of 10 (unless otherwise stated) in order to determine optimum k-mer size for the 76bp reads. Apart from varying k-mer size (word size), default settings were used for all assemblers from our observation that gap / extension costs and bubble size (branch collapse distances) had a marginal effect and were broadly similar at default (data not shown) with 100nt reads.

### Part-Simulated viral metagenomics dataset

62 full-length viral chromatids from 35 distinct human viral genomes were collated. The viruses were chosen to reflect a molecular genetic and life-style spread by choosing them from all Baltimore viral classification groups. Names, accession numbers, and viral groups chosen include:

Alphapapillomavirus_7, group I, NC_001357.1. Human_papillomavirus_type_16, group I, NC_001526.2. Human_herpesvirus_1, group I, NC_001806.1. Vaccinia_ virus, group I, NC_006998.1. Human_herpesvirus_8, group I, NC_009333.1. Merkel_cell_polyomavirus, group I, NC_010277.1. Human_adenovirus_54, group I, NC_012959.1. Adeno-associated_virus_1, group II, NC_002077.1. Human_ parvovirus_B19, group II, NC_000883.2. Torque_teno_virus group II, NC_015783.1. Colorado_tick_fever_virus, group III, NC_004181.1, NC_004182.1, NC_004183.1, NC_004184.1, NC_004185.1, NC_004186.1, NC_004187.1, NC_004188.1, NC_004180.1, NC_004189.1, NC_004191.1, NC_004190.1. Rotavirus_ A, group III, NC_011500.2NC_011501.2, NC_011502.2NC_011503.2, NC_011504.2, NC_011505.2, NC_011506.2, NC_011507.2, NC_011508.2, NC_011509.2, NC_011510.2. Hepatitis_E_virus, group IV, NC_001434.1. Hepatitis_ A_virus, group IV, NC_001489.1. Rubella_virus, group IV, NC_001545.2. Human_ astrovirus, group IV, NC_001943.1. Norwalk_virus, group IV, NC_001959.2. Hepatitis_C_virus, group IV, NC_004102.1. Human_coronavirus_HKU1, group IV, NC_006577.2. Rabies_virus, group V, NC_001542.1. Vesicular_ stomatitis_Indiana_virus, group V, NC_001560.1. Borna_disease_virus, group V, NC_001607.1. Marburg_marburgvirus, group V, NC_001608.3. Mumps_virus, group V, NC_002200.1. Ebola_virus_Mayinga_Zaire_1976, group V, NC_002549.1. Human_metapneumovirus, group V, NC_004148.2. Lassa_virus, group V, NC_004297.1. Influenza_A_virus__A_New_York_392_2004_H3N2, group V, NC_007366.1, NC_007367.1, NC_007368.1, NC_007369.1, NC_007370.1, NC_007371.1, NC_007372.1, NC_007373.1. Rift_Valley_fever_virus, group V, NC_014397.1, NC_001653.2. Human_T-lymphotropic_virus_1, group VI, NC_001436.1. Human_immunodeficiency_virus_1, group VI, NC_001802.1. Human_ immunodeficiency_virus_2, group VI, NC_001722.1. Hepatitis_B_virus, groupVII, NC_003977.1. Hepatitis_delta_virus, group NA, NC_001653.2.

Illumina paired end reads (100nt in length) were simulated using the freely available software, pIRS [41] (ftp://ftp.genomics.org.cn/pub/pIRS/). The healthy (non virally infected) human liver sample total RNA Illumina Hi-Seq FASTQ data set (above) was used to train the software to modify the viral genome sequences. The modifications included percentage GC content profile, error and quality distribution, read size and pair distance mirroring. The viral read sequences were pIRS selected to match the clean liver reads including the mean paired distance at 327.96nt with an SD of 77.1 with the read lengths universally set at 100nt as with the clean liver reads. Additionally, pIRS was used to generate different depths of viral sequence coverage (0.5, 1, 2, 5, 10 and 100x coverage data sets). The different viral coverage data sets were then separately embedded into ~17,200,000 paired-end reads from the healthy liver FASTQ set used to ‘train’ the pIRS software. Optimal word size assembly was determined with the use of CLC Bio assembler v6.1, (Arhus, Denmark) to assemble contigs across a range of word sizes [13, 17, 19, 21, 23, 25, 31, 41, 49]. Optimal word size was determined with all coverage sets by mapping the assembled contigs to the original collated viral genome dataset described using CLC Bio Mapper v6.1, (Arhus, Denmark). The largest viral contigs and total reference coverage of all contigs was optimal at a word size of 21 for all the datasets (0.5, 1, 2, 5, 10 and 100x coverage data sets). Assembled contigs covering >90% of each viral reference required 10x read coverage or greater. Consequently, the 10x coverage set was used to subsequently validate the effects of the k-mer frequency filter (Kontaminant) and the hostmapping subtraction processes. The artificial 10x coverage viral data-set included 70,602 viral paired reads combined with 17.2 x 106 human liver paired reads). Additional data sets used to ascertain percentage host-mapping subtraction by individual reference sets (see Host mapping subtraction details below and Fig 3) were derived from ten idiopathic liver biopsies processed for total RNA and separately for cytosolic viral enriched fractions, as with the HBV and HCV sets [11]. Samples were sequenced with the Illumina Hi-Seq 2500 100 nt paired-end protocol. Average total RNA set read sizes were 190 million paired reads. Average viral enriched cytosolic set read sizes were 82 million paired reads.

### Host mapping subtraction

The artificial, pIRS generated viral metagenomics data set embedded in total RNA derived non-infected liver Illumina paired-end reads was used to determine the percentage subtraction of host and viral sequence reads using BWA, Bowtie and CLC mapping algorithms. BWA-MEM (version 0.7.5a-r405), Bowtie2 (version 2.1.0), CLC Bio v6.5 (Arhus, Denmark).

The CLC mapper was tested by fixing the gap and mismatch penalty at 3 and 2 respectively, and altering the proportion of the sequence read aligned (70–100%) and the homology to the reference sequence of the aligned portion of the sequence read (70–100%). Bowtie and BWA were run in paired-end mode with the scoring settings adjusted to reflect CLC parameters. Mismatch penalty was set to 2, gap open penalty was set to 3 and gap extension was set to 1. All other parameters were set as default. To compare the percentage of read and identity therein, BWA and Bowtie mapped reads were extracted from each Sequence Alignment/Map (SAM) file and a custom Perl script was used to parse the CIGAR and MD tags of each mapped read and subsequently calculate the proportion of the sequence reads aligned and the homology to the reference sequence of the aligned portion of the sequence read.

At ≥80% of the read with ≥90% homology to a human reference sequence, the percentage of viral sequence reads subtracted was 0.016–0.02% depending on the algorithm tested. This setting was used for all subsequent host-mapping subtraction experiments [Figs 2–3 and 12]. The human sequence reference sets included: a) the Genome Reference Consortium Human Build 37 patch release 10 (GRCh37.p10); b) the complete genome of *Homo sapiens* mitochondrion, NC_012920.1; and c) a *Homo sapiens* ribosomal RNA set (18S complete rRNA gene M10098 & X03205, 45S pre-rRNA NR_046235, 5S rRNA NR_023363, and 28S rRNA NR_003287). NB: the mitochondrial consensus is not included in the GRC human build 37 and the four cytoplasmic rRNA molecules (non-MT encoded) are included separately due to the presence of the spacer DNA in the genomic sequence.

### K-mer filtering

K-mer filtering was carried out using the freely available Kontaminant tool developed at The Genome Analysis Centre (TGAC), http://www.tgac.ac.uk/kontaminant/ or https://github.com/TGAC/kontaminant [42]. To use the tool, a k-mer library is first made from a reference–in our case, human reference GRCh37 from the Genome Reference Consortium. The k-mer library is created by sliding a window of size *k* over the reference, base-by-base, creating k-mers as it moves. Reads are then filtered by scanning FASTQ files through Kontaminant and comparing k-mers in reads with the k-mers in the reference. A read is filtered (discarded) if the number of shared k-mers with the reference is greater than or equal to a threshold value (the default, 1 k-mer, was used). A k-mer size of 21 was used. Observations have shown that k-mers of this size tend to be unique amongst different species and should be more than large enough to differentiate between viral and human genomes [50–51]. Multiple idiopathic hepatitis liver sample (100nt Illumina NGS reads) datasets were used to benchmark the use of Kontaminant at >50 million reads/hour/core using a standard multi-core desktop computer with a modern version of unix installed).

### Low complexity filtering:

Low complexity filtering was performed using mdust [47], a standalone version of the DUST module from BLAST (R. Tatusov and D.J. Lipman, unpublished data). DUST was used to mask repetitive / low complexity sequences including short tandem repeats and variable number tandem repeats. Default settings were used: maximum word size of 3 and cutoff of 28. Prior to k-mer filtering, host-mapping subtraction and *de novo* assembly, Illumina FASTQ data was converted to FASTA as input to mdust. Low complexity regions were masked as ambiguous and these regions were subsequently trimmed as described.

### Post-Assembly viral contig analysis

Contig length and total reference coverage of viral contigs were determined for all sets and experiments by the same method irrespective of the read filtering method used pre-assembly: 1) Assembled consensus contigs were extracted in FASTA format unless otherwise stated. 2) These contigs were aligned to a database containing the viral references and the human reference using BLAST+ 2.2.25. The blastn program was used with default settings. To ascertain if there were any chimeric contigs, all BLAST hits were examined for each contig. If a contig partially aligned with 75% identity to both the human reference and a viral reference, it was flagged as chimeric. No such contigs were found for any of the assemblers. 3) Non-chimeric contigs matching the viral references were extracted as a FASTA file and mapped to the references. This process allowed common reference contigs to be overlaid on their respective reference sequences to ascertain a) the total reference coverage, b) the coverage of the largest contig, c) to observe the degree of consensus identity to the reference and to discount contig terminal end mismatching from the coverage and contig size estimations. The nucleotide sequences of all the resulting viral contigs deviated from the reference by less than 1%. Standard N25-N90 values used (defined as the smallest contig of a minimal set of contigs required to achieve coverage of the viral reference indicated, expressed as a % of the reference coverage). Fig 10B) shows the arithmetic mean and SD derived from these values for each experimental set.

### Idiopathic contig BLAST settings and filtering parameters

Two idiopathic hepatitis liver samples (Total RNA Illumina NGS sequenced) were pre-assembly filtered with a short read mapper and a K-mer filter as previously described [Fig 12A.]. Remaining reads were optimally assembled (as previously described) and the assembled contig numbers are shown in Fig 12B. Contigs were subsequently filtered for putative viral sequences using BLASTn and tBLASTx (blast-2.2.28.mt) to a complete nt database (NCBI nt). All blast settings default except for blastn:-max_target_seqs 50-evalue 0.0001-dust yes; tblastx:-num_alignments 50-evalue 0.0001-seg yes. Output was parsed for our filtering criteria: e.value, greatest hit length, greatest identity, percentage of query sequence in greatest hit, taxonomic descriptors. Parsed data was subsequently filtered for putative viral hit lengths > 300nt, ID > 85%, % query in hit > 90%, E.value < 10^−80^. Unique query hits were then selected by highest hit length. Contig queries were retained if viral taxonomic descriptors matched for all the above criteria (by viral family). Matching query sequences from each sample were collated and mapped to the best-hit reference to determine percentage coverage of each contig and total coverage of the reference [Fig 12C.]. Each of the idiopathic samples showed viral hits to two distinct viral families only (U42720, AY232737 and AF345527, AJ507799 respectively). Smaller contigs outside of the filtering strategy employed were subsequently categorised by aligning all the assembled contigs to the references to capture sub-300nt contigs with >90% homology over 90% of the contig with the contig lower limit of 101nt. No additional contigs were found in the unfiltered assembled contig set that were not present in the mapper and k-mer filtered experimental sets.

### SURPI pipeline comparison

As described above, the two HCV infected liver datasets with 0.7x and 9x mean depth of viral genome coverage and the metagenomics viral dataset embedded in Illumina 100nt paired-end clean liver reads were used to compare the SURPI (comprehensive mode) pipeline [9] output to our processes. Viral read fastq headers were modified to include the viral chromosome/chromatid identities for subsequent characterization. *De novo* assembly files were taken from the default output directory and pre-processing and subtraction step information was extracted from the human.snap.unmatched / preprocessed and cutadapt.cropped.dusted.bad FASTQ default output files.

SURPI (http://chiulab.ucsf.edu/surpi/) was installed as an Amazon EC2 cloud-computing instance using default parameters and all dependencies installed.
